# Novel Dominant Splicing Variant in *MPZ* Associated With Unusual Charcot–Marie–Tooth Disease

**DOI:** 10.1111/jns.70085

**Published:** 2025-12-09

**Authors:** Anthony Maino, Florence Hazane‐Puch, Philippe Petiot, Nathalie Roux‐Buisson, John Rendu, Julien Fauré, Gaëlle Hardy

**Affiliations:** ^1^ CHU Grenoble Alpes, Laboratoire de Biochimie et Génétique Moléculaire Grenoble France; ^2^ Université Grenoble Alpes, Inserm, U1216, CHU Grenoble Alpes, Grenoble Institut Neurosciences Grenoble France; ^3^ Service d'Electroneuromyographie et de Pathologies Neuromusculaires, Hôpital Neurologique Pierre Wertheimer Lyon France

**Keywords:** Charcot–Marie–Tooth, *MPZ*, myelin P0 protein, splice variant

## Abstract

**Background and Aims:**

Variants in the myelin protein zero coding *MPZ* gene are responsible for a broad spectrum of peripheral demyelinating and axonal neuropathies, including different types of Charcot–Marie–Tooth diseases, challenging for genotype–phenotype correlation.

**Methods:**

Minigene splicing reporter assay was used to unveil the pathogenic mechanism of a novel *MPZ*(NM_000530.8) c.234 + 1G>C p.(?) heterozygous splice variant.

**Results:**

The variant was identified in a 47‐year‐old female patient presenting with atypical clinical features, including balance disturbance with positive Romberg, absent Achilles tendon reflexes, distal hypoesthesia and bulbar involvement, including dysarthria and dysphagia. Electromyography revealed a sensory‐motor neuropathy with moderately reduced nerve conduction velocities. In silico analysis predicted this variant to disrupt the consensual donor splice site located in intron 2 of *MPZ*. Minigene construction confirmed the functional impact of this variant, revealing exon 2 skipping and the apparition of a premature termination codon.

**Interpretation:**

This case expends the genotype–phenotype correlations of *MPZ*‐related Charcot–Marie–Tooth diseases, associating atypical mild phenotype with a rare splice dominant variant, and provides new insights into *MPZ* haploinsufficiency and pathogenic mechanisms.

## 1 Introduction

Myelin protein zero (MPZ), also known as P_0_, is a major structural component of the peripheral nervous system (PNS) myelin, accounting for 50% to 60% of the proteins in this tissue. Specifically expressed in Schwann cells, this transmembrane glycoprotein is critical for myelination by compacting myelin wraps through homotypic interactions between extracellular domains, acting similarly as an immunoglobulin adhesion molecule [1, 2]. Pathogenic variants in the *MPZ* gene have been associated with various subtypes of demyelinating and axonal peripheral neuropathies, including Charcot–Marie–Tooth (CMT) disease (types 1B, 2I, and 2J), juvenile Dejerine‐Sottas disease or Roussy‐Levy syndrome. The clinical spectrum of *MPZ*‐related neuropathies is highly heterogeneous, ranging from mild to severe forms, early to late onset, and various degrees of nerve conduction velocity (NCV) impairment. Inheritance is almost exclusively autosomal dominant although half of reported cases are sporadic [3, 4]. However, the underlying pathological mechanisms remain incompletely understood as both gain‐ and loss‐of‐functions have been shown to cause the disease [5, 6, 7]. Most pathogenic *MPZ* variants are distributed across the extracellular domain, but variants in the cytoplasmic domain have also been identified. The majority of variants identified are truncating and missense variants, and splice variants remain relatively rare. We report here a novel dominant heterozygous splice variant *MPZ*:c.234 + 1G>A with unusual CMT manifestations. Minigene construction demonstrated that this variant was responsible for an exon 2 skipping, which should lead to the apparition of an early premature termination codon p.(Val23Aspfs*12).

## 2 Materials and Methods

Molecular genetics. Patient's DNA was extracted from whole blood sample using QIAsymphony instrument (Qiagen). DNA concentration and quality were assessed by absorbance measurement (Nanodrop, Thermofisher). Exome sequencing was performed by next‐generation sequencing (NGS) using Agilent Sureselect Human All Exon v8 kit on Illumina Seq500. Alignment and data analysis were performed using SeqOne v1.2. Sanger sequencing was performed on ABI3500XL (Thermofisher) with the following primers: 2F‐ CCTCTGACCTGTGACTCAGC and 2R‐GGTAAGGGATCTTGGGCTGG.

Minigene splicing reporter assay. DNA from the patient carrying the identified heterozygous splice *MPZ*‐variant was amplified using CloneAmp HiFi PCR Premix (#639298, Takara) with the following primer sequences (5′‐3′): forward (intron 1): CTAAACAGCCACATATGTCTTGGAAGGGAATACAGGAAAGG and, reverse (intron 2): CCCCCCTCGACCATATGATCTTGGGCTGGAAAGGG. The 835 bp amplicon (encompassing entire intron 1, exon 2 and intron 2) was inserted in the NdeI restriction site of the pTB2 minigene vector using In‐Fusion HD Cloning Kit (#102518, Takara). Constructed vectors were transformed in *Escherichia coli* DH5α‐T1R One Shot MAX Efficiency (#12297016, ThermoFisher) for amplification. Clones were checked after digestion by NdeI (#ER0581, ThermoFisher) and then confirmed by Sanger sequencing. Plasmids containing WT or mutated *MPZ* sequences were then chosen. HEK293T cells were purchased from the American Type Culture Collection (ATCC, USA) and grown in DMEM medium (#31966021, ThermoFisher) supplemented with 10% fetal bovine serum (#10270106, ThermoFisher), penicillin–streptomycin (respectively 100 U/L and 100 mg/L, #15140122, ThermoFisher). Cell cultures were incubated at 37°C and 5% CO_2_ in a humidified incubator. The day before transfection, 2.10^5^ cells were plated in a six‐well culture plate and transiently transfected with 1 μg of plasmids (empty pTB2 (control), pTB2‐WT (WT) and pTB2‐mutant) using calcium phosphate (#631312, Takara) according to the manufacturer's instructions. After 48 h of incubation, cells were harvested with trypsin–EDTA (#15400054, ThermoFisher) and total RNA was extracted (NucleoSpin RNA, #740955.50, Macherey Nagel). First‐strand cDNA was synthesized from 1 μg of extracted total RNA (SuperScript III First‐Strand Synthesis SuperMix, #11752050, ThermoFisherScientific). The resulting cDNA was amplified by PCR using vector‐specific primers surrounding the cloning site thanks to CloneAmp HiFi PCR Premix (#639298, Takara). The PCR products were analyzed on a 1% agarose gel. The target DNA bands were gel‐cut, purified (PCR Clean up, #740609‐250, Macherey Nagel) and sequenced to identify mutation impact on the splicing process. All the DNA sequences of amplicons cloned into the pTB2 vector, WT and mutant plasmids were checked by DNA sequencing (3500xl Genetic Analyzer, Thermo Fisher Scientific, and SnapGene software).

## 3 Results

### 3.1 Clinical Presentation

A 47‐year‐old woman presented with a three‐year history of progressive and worsening gait imbalance. Symptoms oriented towards a sensory neuropathy with no evidence of motor weakness. An initial working diagnosis of chronic inflammatory demyelinating polyradiculoneuropathy (CIDP) was considered, but intravenous immunoglobulin treatment was ineffective. Cerebrospinal fluid analysis was unremarkable, and inflammatory or autoimmune etiologies were also excluded.

Neurological examination revealed marked signs of ataxia, with a predominant sensory component, a positive Romberg sign, and absent Achilles tendon reflexes. Tandem gait was unachievable. A cerebellar component could not be excluded, given the presence of heel‐to‐knee dysmetria in the eyes‐open condition, and a subtle lateral jerk nystagmus. The finger‐to‐nose testing was relatively preserved. There was no ophthalmoparesis, ptosis, or other oculomotor abnormality. Additional findings included distal hypoesthesia in a stocking distribution, extending from the ankles to the feet. Electromyography study demonstrated consistent findings of a sensorimotor demyelinating polyneuropathy with reduced nerve conduction velocity (NCV) values affecting both proximal and distal locations (Table S1).

In addition, the patient exhibited atypical bulbar manifestations while brain MRI was unremarkable. These manifestations included dysarthria, dysphonia, and dysphagia with episodes of aspiration but no evidence of lingual atrophy. Given the coexistence of sensory neuropathy and bulbar dysfunction, further testing for anti‐ganglioside antibodies was conducted to exclude CANOMAD syndrome and returned negative. Genetic analysis of the *POLG* gene to exclude SANDO syndrome, screening for spinocerebellar ataxias, metabolic evaluation for adrenoleukodystrophy, antibody panels for nodopathies, serum protein electrophoresis, and antineuronal antibody panels were all negative.

Assuming the hypothesis of an uncharacterized immune‐mediated neuropathy, corticosteroid therapy was initiated and well tolerated. This regimen resulted in a transient improvement of sensory disturbance in the left upper limb, but no sustained benefit was observed on gait or global ataxia. Given the failure of previous treatments, plasma exchange sessions were attempted as a therapeutic approach for CIDP, but this intervention also proved ineffective. In light of the refractoriness to immunomodulatory therapies, the patient was ultimately managed with supportive physiotherapy and speech therapy.

### 3.2 Molecular Analysis and Minigene Splicing Reporter Assay

**FIGURE 1:**
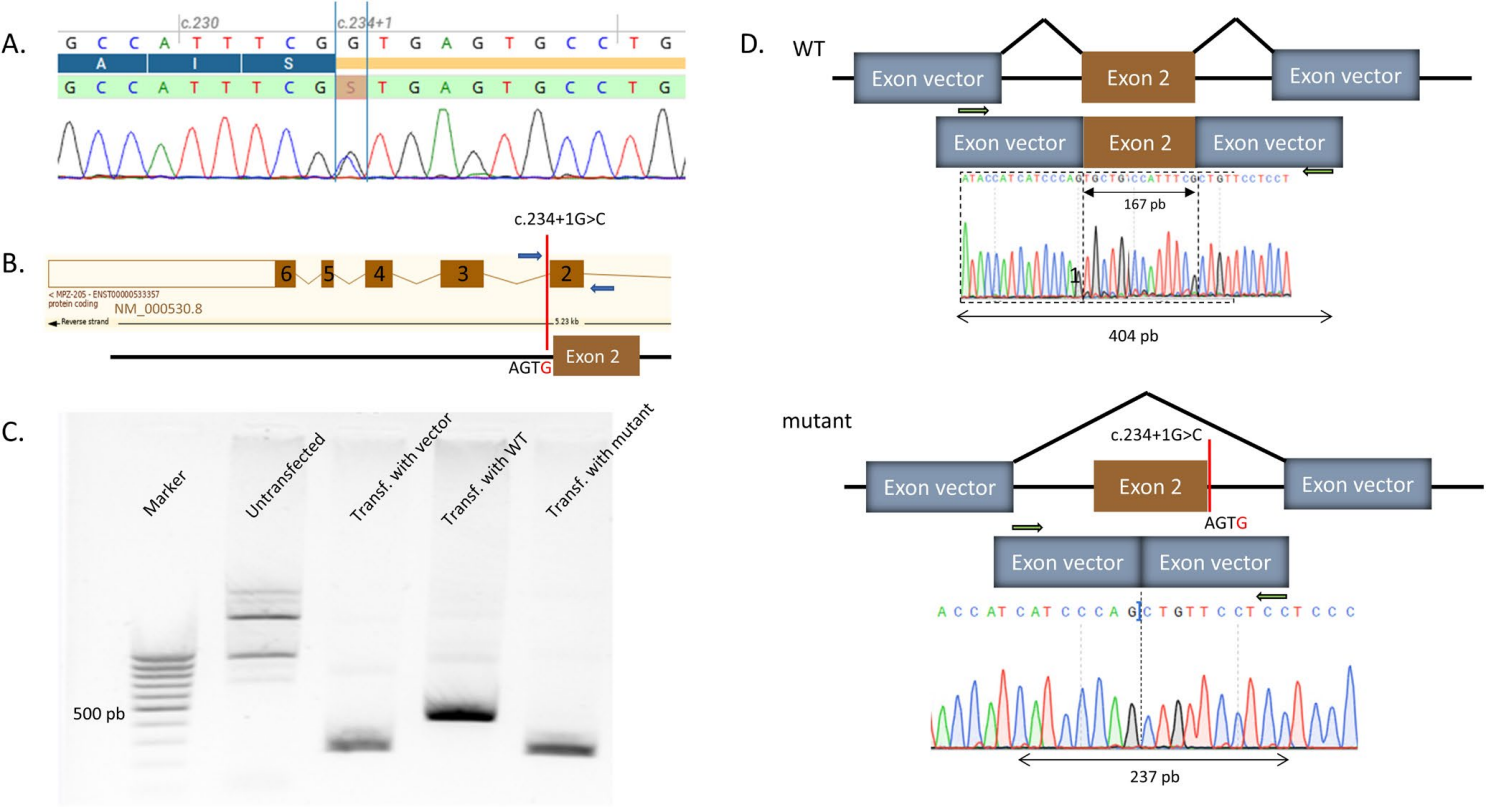
Minigene splicing reporter assay. (A) Sanger sequencing on DNA. Position c.234 + 1 is highlighted by the blue bars. The S symbol corresponds to the heterozygous C>G transition to IUPAC nomenclature. Sequences generated from ABI3500XL (Thermofisher) are visualized using Alamut Visual Plus version v1.8.1 (©2023 SOPHiA GENETICS). (B) Minigene construction. The strategy aimed to amplify the genomic MPZ sequence bordered by introns 1 and 2 in the patient. The primer set (blue arrows) enabled amplification of 475 and 193 bp of introns 1 and 2, respectively, along with the entire sequences of exons 2 (167 bp). (C) Gel electrophoresis UV visualization of RT‐PCR products for each condition. (D) Sanger sequencing on cDNA from minigene vectors. Sequencing with primer sets on exon vectors (green arrows) experiments revealed normal splicing in HEK293T cells transfected with the wild‐type (WT) MPZ minigene (top). Conversely, cells transfected with the mutant minigene showed an exon 2 skipping (bottom).

Whole exome sequencing identified a novel heterozygous *MPZ*(NM_000530.8) c.234 + 1G>C p.(?) variant. This variant was further confirmed by Sanger sequencing (Figure 1A). This variant is absent from the GnomAD v.4.1 database and is predicted to be deleterious by several bioinformatics algorithms with splicing predictions indicating a loss of the consensus donor site in exon 2 (MaxEntScan, SPiP, SpliceAI; see Table S2). In silico splicing predictions were confirmed using a minigene construction encompassing the variant position (Figure 1B). Using a pTB2 vector, HEK293T cells were transfected with the plasmids under three conditions: empty pTB2 (control), pTB2‐wild‐type (WT) and pTB2‐mutant. Untransfected HEK293T cells were also used as controls. RNA was extracted after 48 h incubation and was sequenced after retro‐transcription. PCR product sizes corresponded to the addition of 167 bp from exon 2 when comparing WT to mutant (Figure 1C). Sanger sequencing confirmed a complete skipping of the inserted *MPZ*‐exon 2 in the mutated condition (Figure 1D). As a consequence, the exon 2 skipping should lead to a shift in the reading frame and to the apparition of an early premature termination codon p.(Val23Aspfs*12).

## 4 Discussion

Pathogenic variants in the *MPZ* gene are associated with a broad spectrum of inherited peripheral neuropathies, primarily characterized by progressive muscle weakness, atrophy, and sensory loss. These manifestations result from either peripheral nerve demyelination or axonal degeneration [8]. However, establishing the clinical significance of newly discovered *MPZ* variants can be challenging due to this phenotypic heterogeneity across patients. It has been suggested that early‐onset phenotypes are linked to impaired developmental myelination, as evidenced by nerve biopsies, whereas adult forms are thought to result from progressive axonal degeneration [8]. Physiopathological pathways are complex, including both gain‐ and loss‐of‐function. Haploinsufficiency has been suggested to be pathogenic in humans, but usually with a mild late‐onset phenotype [7, 9]. Animal models corroborate these observations as heterozygous null mice have been shown to produce half as much *MPZ* mRNA and reduced protein levels compared to wild‐type mice [10]. While these mice undergo a normal myelination process during development, they develop late‐onset progressive demyelination accompanied by moderate electrophysiological degradation, consistent with the observations made in CMT1B patients [11]. Conversely, other studies have shown that *MPZ* overexpression led to congenital myelination defects, suggesting a distinct pathogenic mechanism [12].

We report here the case of a 47‐year‐old female patient with slow conduction velocities in motor and sensory nerves showing uncommon bulbar features challenging for the diagnosis of CMT. It is yet unclear whether these bulbar and cerebellar features are associated with CMT as no other cause was identified and central MRI findings were not informative. Interestingly, dysphagia was reported in one French family diagnosed with CMT due to a pathogenic variant in the *MPZ* gene but this case involved a severe phenotype including hearing loss and Argyll Robertson‐like pupils [13].

In the present case, the patient's complex diagnosis journey concluded with whole exome sequencing, revealing a novel heterozygous splice variant c.234 + 1G>C in the *MPZ* gene. No familial history was reported but no parents nor relatives were available for genetic testing to assess for the inheritance of this variant. Insufficient evidence was then available for classification besides in silico tools that were all in favor of a deleterious effect with a major splicing defect. Strikingly, this case was very similar to a recently reported case by Terkelsen et al. [14] of a 61‐year‐old female patient, bearing the c.234 + 1G>A variant in the heterozygous state at the same site. The patient had clear signs of demyelination in nerve conduction electrophysiological examination and a predominantly distal phenotype with *pes cavus*, wiggling feet, foot muscle atrophy, marked toe weakness and Achilles tendon reflex abolition. The patient also presented a mild loss of sensation from the mid‐calf to the distal level. This variant c.234 + 1G>A had already been reported once in 2020 by Tanigushi et al. in a Japanese patient published a study reporting a large review of *MPZ* variants in Japanese patients presenting signs of demyelinating CMT with severe weakness of all limbs, but this variant remained classified as a variant of unknown significance [15]. Terkelsen et al. further demonstrated the protein impact of this variant, showing that it was responsible for exon 2 skipping using CRISPR activation (CRISPRa) on healthy donors' skin fibroblasts. Following induced *MPZ* expression in these cells, transcript long‐read sequencing demonstrated the complete exon 2 skipping resulting in a premature termination codon p.(Val23Aspfs*12). Nonsense‐mediated mRNA decay (NMD) was confirmed using cycloheximide treatment [14].

Based on similar clinical presentations between our patient and the patient described by Terkelsen et al. (Table S2), and based on consistent bioinformatic predictions (Table S3), we hypothesized that the c.234 + 1G>C variant should result in an analogous splicing defect. Minigene construction in both wild‐type and mutant conditions confirmed a similar scenario with complete exon 2 skipping. Finally, we were able to classify this c.234 + 1G>C variant as likely pathogenic based on this functional evidence.

In conclusion, this case describes atypical features of *MPZ*‐related neuropathy, notably the unusual bulbar involvement with dysarthria, associated with a rare splice variant disrupting the consensus donor site in intron 2 of *MPZ*. We highlight here the relevance of a minigene construction to assess the impact of splice variants when native tissue mRNA is difficult to sample, as demonstrated before in this context [16, 17, 18]. Indeed, *MPZ* expression is not ubiquitous and is only expressed in PNS cells. This strategy could be of particular interest for elucidating the pathogenicity of new splice variants. These findings provide new elements for genotype–phenotype correlation in *MPZ*‐related disorders and support that the heterozygous splice variant leading to a premature termination codon should be pathogenic by haploinsufficiency when subjected to NMD. Although no specific treatments are currently available for *MPZ*‐related neuropathies, proving an accurate genetic etiology to patients is essential as emerging therapies are being experimented with in this context [19, 20, 21].

## Conflicts of Interest

The authors declare no conflicts of interest.

## Supporting information


**Data S1:** jns70085‐sup‐0001‐Supinfo.docx.

## Data Availability

The data that support the findings of this study are available from the corresponding author upon reasonable request.
