# Supplementary material for: Novel Dominant Splicing Variant in MPZ Associated With Unusual Charcot–Marie–Tooth Disease
Source: J Peripher Nerv Syst. 2025 Dec 9;30(4):e70085. doi: 10.1111/jns.70085 (PMC12687107; doi:10.1111/jns.70085)
Supplement: Supplementary file 1 — Data S1: jns70085‐sup‐0001‐Supinfo.docx. [file JNS-30-0-s001.docx]

**Supp Table 1. Nerve Conduction Study results**

| **MOTOR CONDUCTION STUDY** | | | | | | | | | | | | |
| --- | --- | --- | --- | --- | --- | --- | --- | --- | --- | --- | --- | --- |
| Site | Latency  (ms) | | CMAP Amplitude (mV) | Area (mV/ms) | | Duration (ms) | Distance (mm) | | Conduction Velocity (m/s) | Area Ratio (%) | | F-wave Latency (ms) |
| **Motor Median nerve (left)** | | | | | | | | | | | | |
| Wrist - *ABP* \| *ABP* | 4.21 | | 9.6 | 29.3 | | 5.4 |  | |  |  | | 34.7 |
| Elbow-Wrist \| *ABP* | 9.81 | | 9.6 | 29.3 | | 5.6 | 230 | | 41.1 | 0 | |  |
| **Motor Median nerve (right)** | | | | | | | | | | | | |
| Wrist - *ABP* \| *ABP* | 4.58 | | 6.9 | 19.4 | | 4.8 |  | |  |  | | 35.3 |
| Elbow-Wrist \| *ABP* | 9.85 | | 6.7 | 20.6 | | 5.4 | 215 | | 40.8 | 6.2 | |  |
| **Motor External Popliteal Sciatica nerve (left)** | | | | | | | | | | | | |
| Sub-neck of the fibula - Tib. ant \| Tib. ant. | 3.67 | | 4.7 | 24.0 | | 9.1 |  | |  |  | |  |
| Sus-neck – Sub-neck of the fibula \| Tib. ant. | 5.29 | | 4.8 | 25.0 | | 10.0 | 80.0 | | 49.4 | 4.2 | |  |
| Ankle-Foot \| Foot | 6.99 | | 0.11 | 0.28 | | 6.5 | 335 | |  |  | |  |
| Sub-ankle \| Foot | 18.5 | | 0.19 | 0.76 | | 8.7 | 335 | | 29.1 | 171 | |  |
| **Motor External Popliteal Sciatica nerve (right)** | | | | | | | | | | | | |
| Ankle-Foot \| Foot | 6.44 | | 3.3 | 12.2 | | 7.2 |  | |  |  | |  |
| Sub-ankle \| Foot | 16.9 | | 2.9 | 12.1 | | 8.0 | 310 | | 29.6 | -0.82 | |  |
| **Motor Internal Popliteal Sciatica nerve (left)** | | | | | | | | | | | | |
| Ankle-FHB \| FHB | 10.4 | | 9.8 | 32.3 | | 6.5 |  | |  |  | | 75.8 |
| Popliteal fossa-ankle \| FHB | 23.6 | | 8.4 | 30.9 | | 7.4 | 400 | | 30.3 | -4.3 | |  |
| **Motor Internal Popliteal Sciatica nerve (right)** | | | | | | | | | | | | |
| Ankle-FHB \| FHB | 6.80 | | 11.1 | 29.3 | | 9.0 |  | |  |  | | 71.7 |
| Popliteal fossa-ankle \| FHB | 18.4 | | 8.0 | 29.1 | | 11.0 | 425 | | 36.6 | -0.68 | |  |
| **Motor Ulnar nerve (left)** | | | | | | | | | | | | |
| Wrist - ADM\| ADM | 4.04 | | 6.7 | 25.7 | | 5.8 |  | |  |  | |  |
| Sub-elbow - wrist \| ADM | 9.42 | | 7.1 | 24.8 | | 5.8 | 250 | | 46.5 | -3.5 | |  |
| **Motor Ulnar nerve (right)** | | | | | | | | | | | | |
| Wrist - ADM\| ADM | 3.04 | | 7.4 | 28.3 | | 6.2 |  | |  |  | | 35.5 |
| Sub-elbow - wrist \| ADM | 8.21 | | 6.8 | 27.4 | | 6.5 | 220 | | 42.6 | -3.2 | |  |
| **SENSORY CONDUCTION STUDY** | | | | | | | | | | | | |
| Sites | | Latency  (ms) | | | SNAP Amplitude (µV) | | | Conduction Velocity (m/s) | | | Distance  (mm) | |
| **Sensory Median nerve (right)** | | | | | | | | | | | | |
| Wrist - Digit III | | 3.59 | | | 17.7 | | | 36.2 | | | 130 | |
| **Sensory Radial nerve (right)** | | | | | | | | | | | | |
| Forearm - Wrist | | 2.38 | | | 10.5 | | | 31.5 | | | 75.0 | |
| Forearm - Wrist \| Wrist | |  | | |  | | | 31.5 | | | 75.0 | |
| **Sensory Sural (external saphenous) nerve (left)** | | | | | | | | | | | | |
| Mid-leg - Malleolus | | 3.47 | | | 9.4 | | | 27.4 | | | 95.0 | |
| **Sensory Sural (external saphenous) nerve (right)** | | | | | | | | | | | | |
| Mid-leg - Malleolus | | 3.72 | | | 5.6 | | | 28.2 | | | 105 | |
| **Ulnaire Sensitif Droit** | | | | | | | | | | | | |
| Wrist - Digit V | | 3.06 | | | 16.0 | | | 34.3 | | | 105 | |

ABP = abductor pollicis brevis; Tib. ant. = tibialis anterior; FHB = flexor hallucis brevis; ADM = abductor digiti minimi

**Supp Table 2. Phenotypic description and electrophysiological features of the two patients*.***

|  |  | **Present case** | **Terkelsen *et al*** (13) |
| --- | --- | --- | --- |
| Gender |  | Female | Female |
| Age at diagnosis |  | 47 | 61 |
| History |  | Progressive | Progressive |
| Motricity |  | No motor deficiency | Distal muscle weakness ankle/toe |
| Reflexes |  | Achilles tendon reflex abolition | Achilles tendon reflex abolition |
| Signs of ataxia |  | Balance trouble, Romberg sign  Dissymmetry heel-knee |  |
| Sensitivity |  | No sensitivity trouble in extremities  Hypoaesthesia in ankle and feet | Loss of sensation from the mid-calf level and distally |
| Other phenotypic features |  | Dysarthria, dysphagia, dysphonia | Pes cavus |
| Electromyogram examination |  | Demyelinating profile with signs of sensitivomotor neuropathy | Reduction in motor and sensitive nerve conduction velocities |

**Supp Table 3. Comparison of *MPZ*:c.234+1G>C and *MPZ*:c.234+1G>A variants.**

|  | **c.234+1G>C** | **c.234+1G>A** |
| --- | --- | --- |
| GnomAD v.4 allele count | 0 | 1 |
| MPA impact | High splice | High splice |
| MaxEntScan 5'ss scores  *(%variation Ref vs Alt)* | -74.44% | -73.63% |
| dbscSNV ADA score | 1.00 | 1.00 |
| SpliceAI Donor Loss score | 1.00 | 0.98 |
| SPiP | 98.41% | 98.41% |
| Pangolin splice loss | 0.87 | 0.87 |
| BayesDel addAF v.4.6 | 0.5832 | 0.5832 |
| EIGEN | 1.0647 | 1.0647 |
| Protein impact and method of assessment | p.(Val23Aspfs12*)  assessed with minigene | p.(Val23Aspfs12*)  assessed with CRISPRa |
